# Cystic Parathyroid Adenomas as a Risk Factor for Severe Hypercalcemia

**DOI:** 10.3390/jcm12154939

**Published:** 2023-07-27

**Authors:** Monika Kaszczewska, Witold Chudziński, Piotr Kaszczewski, Michał Popow, Jakub Grzybowski, Anna Skowrońska-Szcześniak, Herbert Kozubek, Zbigniew Gałązka

**Affiliations:** 1 Department of General, Vascular, Endocrine and Transplantation Surgery, Medical University of Warsaw, 02-097 Warsaw, Poland; monika.choroszy@wp.pl (M.K.); herbert.kozubek@gmail.com (H.K.); zbigniew.galazka@wum.edu.pl (Z.G.); 2 Department of Endocrinology and Internal Medicine, Medical University of Warsaw, 02-097 Warsaw, Poland; ep08@interia.pl (M.P.); anna.skowronskaaa@gmail.com (A.S.-S.); 3 Department of Pathology, Medical University of Warsaw, 02-097 Warsaw, Poland; kuba.grzybowski@wp.pl

**Keywords:** parathyroid cyst, cystic parathyroid adenoma, primary hyperparathyroidism, severe hypercalcemia, hypercalcemic crisis

## Abstract

(1) Background: Parathyroid cystic adenomas (PCA) are rare entities representing only 0.5–1% of parathyroid adenomas, accounting for 1–2% of cases of primary hyperparathyroidism (PHPT). The purpose of this study was to compare classical and functional/secreting cystic parathyroid lesions and identify risk factors for severe hypercalcemia; (2) Methods: A total of 17 patients with PHPT and parathyroid cysts (study group) were compared with the group of 100 patients with hyperparathyroidism caused by adenoma or hyperplasia (control group). In both groups the majority were women (88% vs. 12%, with gender ratio 7, 3:1). The patients were examined preoperatively and postoperatively: PTH, creatine, calcium and phosphate serum and urine concentrations and calcidiol serum levels were assessed; (3) Results: Patients with parathyroid cyst had statistically higher PTH and calcium serum concentration, higher calciuria and lower serum phosphate concentration. There were no statistically significant differences in the concentration of creatine in serum and urine and tubular reabsorption of phosphorus (TRP); (4) Conclusions: Due to higher PTH and calcium levels, cystic parathyroid adenomas could be one of the rare risk factors for severe hypercalcemia and hypercalcemic crisis which can be life threatening.

## 1. Introduction

Primary hyperparathyroidism (PHPT) is a common endocrinological pathology, caused by the overproduction of parathyroid hormone (PTH). It is defined as chronic increase in calcium serum concentration (greater than 2.6 mmol/L, 10.5 mg/dL or ionized calcium greater than 1.25 mmol/L, 5.0 mg/dL). The most common cause of the PHPT (up to 85%) is a parathyroid adenoma (PA). Other reasons include multiple gland involvement (diffuse hyperplasia or multiple adenomas) and parathyroid carcinoma (PC)—accounting for 15% and less than 1%, respectively [1,2,3,4,5].

PHPT affects between 0.1 and 0.3% of the general population, more frequently women, with a gender ratio of 3:1. The disease prevalence increases with age. PHPT is most common in the fifth or sixth decade of life [2,4].

Sporadic PHPT is encountered in around 95% of cases without a known etiological factor. Nonetheless, occasionally in seemingly sporadic cases, some genetic alterations, such as mutations in genes (*cyclin D1*, *RET*) or in tumor suppressor genes (*MEN1*, *CDC73*—formerly *HRPT2*), have been reported.

Familial forms occur in 5% of cases and they include multiple endocrine neoplasia type 1 and 2A (MEN 1, MEN 2A), primary hyperparathyroidism-jaw tumor syndrome (PHPT-JT) and familial hypocalciuric hypercalcemia (FHH) [2].

Nowadays, due to routine serum calcium measurements, most patients do not present any symptoms or have a nonspecific manifestation of the disease such as: tiredness, dysphoria and cognitive impairment. The risk of symptomatic hypercalcemia increases with the duration of the disease. In the course of the disease, renal involvement (nephrolithiasis and renal failure), bone involvement (osteitis fibrosa cystica, osteoporosis, chondrocalcinosis), gastrointestinal disorders (nausea/vomiting, peptic ulcer disease, pancreatitis) and general symptoms (polyuria, polydipsia, abdominal pain, muscle weakness, confusion, coma and cardiac arrest) may occur [1,2,5].

Parathyroid cysts occur rarely, representing 0.5–1% of parathyroid lesions [6,7]. The first information concerning this condition dates back to 1880, and since then, less than 400 cases have been reported [7,8,9].

Only 1–2% of PHPT cases may be caused by cystic degeneration of parathyroid adenoma [10]. Based on the presence or absence of increased PTH serum concentration, parathyroid cysts are categorized as functional or secreting (hemorrhage or cystic degeneration of a parathyroid adenoma), and non-functional or non-secreting (true) parathyroid cysts (from embryologic remnants, a coalescence of microcysts or the result of abnormal retention of PTH). The incidence of both types of parathyroid cysts varied significantly with functional cysts accounting for 10–90% [3,11,12,13]. Functional parathyroid cysts cause symptoms of PHPT (fatigue, depression, osteoporosis, nephrolithiasis) while first manifestation of non-functional cysts may be local mass effect (dysphagia, dyspnea, hoarseness). Cysts are often found accidentally [11,14].

The aim of our study was to compare patients with hyperparathyroidism caused by cystic and solid parathyroid lesions, identifying risk factors for severe hypercalcemia.

## 2. Materials and Methods

The study has been conducted under the approval of the Medical University of Warsaw Bioethical Committee (AKBE/170/2023).

The retrospective study was performed in a group of 117 patients with PHPT admitted to the Department of General, Vascular, Endocrine and Transplantation Surgery, Medical University of Warsaw between 2018 to 2022.

From this group, 17 patients (study group) with primary hyperparathyroidism and cystic lesions were selected.

Each surgery was performed by the same, experienced surgical team. Histopathological examination was carried out postoperatively. The detailed data concerning study and control groups are presented in the Table 1.

Inclusion criteria to the study group:

- primary hyperparathyroidism
- parathormone related hypercalcemia
- parathyroid cyst
- patients after parathyroidectomy

Exclusion criteria from the study group:

- secondary or tertiary hyperparathyroidism
- Inclusion criteria to the control group
- primary hyperparathyroidism
- parathormone related hypercalcemia
- patients after parathyroidectomy

Exclusion criteria from the control group:

- secondary or tertiary hyperparathyroidism

In all study participants the following parameters were assessed: age, volume of the parathyroid gland, pre and postoperative PTH serum concentration, total and ionized preoperative serum calcium concentration, calcinuria, preoperative serum phosphate concentration, preoperative calcidiol concentration, serum creatine concentration, creatinine in the urine sample, GFR and tubular reabsorption of phosphorus—TRP.

Serum and urine levels of calcium, phosphorus and creatinine were measured in the hospital laboratory 1–2 days before and 1–2 days after the surgery. PTH was assessed just after the anesthesia was induced and 15 min after the procedure.

The volume of the removed glands was precisely measured using Archimedes’ principle—each parathyroid gland was placed in a measuring cup filled with water, then the volume of displaced liquid was noted, which was the exact volume of the gland.

In order to obtain microscope slides, sections of 3–5 µm thickness were cut from formalin-fixed paraffin-embedded tissue blocks with a microtome (HM 340E Electronic Rotary Microtome, Thermo Shandon, Waltham, MA, USA). All sections were stained with hematoxylin and eosin (H&E) in an automatic tissue processor (ASP 6026, Leica, Wetzlar, Germany) and then assessed by a qualified pathologist using a light microscope (Nikon Eclipse Ci, Tokyo, Japan).

Statistical analysis was performed with Statistica 13 (StatSoft Polska Sp. z.o.o., Krakow, Poland). The *t*-test, and Mann–Whitney U test were performed. A *t*-test was applied when the normal distribution of data was stated. The Shapiro–Wilk test was performed as a test of normality: a data set with a *p* value of less than 0.05 rejects the null hypothesis that the data are from a normally distributed population. Consecutively, Levene’s test was used to assess the equality of variances—the *p*-value below 0.05 rejects the null hypothesis of equal variances. The normal distribution of data with equal variances was a prerequisite to use the *t*-test. When the normal distribution of data with no equality of variances was observed, the *t*-test with Cochran–Cox correction was performed. When one of the variables was not from a normal distribution the non-parametric Mann–Whitney U test was performed. A regression analysis was also performed.

## 3. Results

Histopathological examination revealed that the cause of the hyperparathyroidism was an adenoma (in 11 cases) or hyperplasia (in 6 patients). In the control group (100 patients) adenomas were diagnosed in 60 cases while hyperplasia was in 40 patients.

### 3.1. No statistical Differences between the Groups with Non-Cystic and Cystic Lesions

#### 3.1.1. Age of the Patients

**Figure 1:**
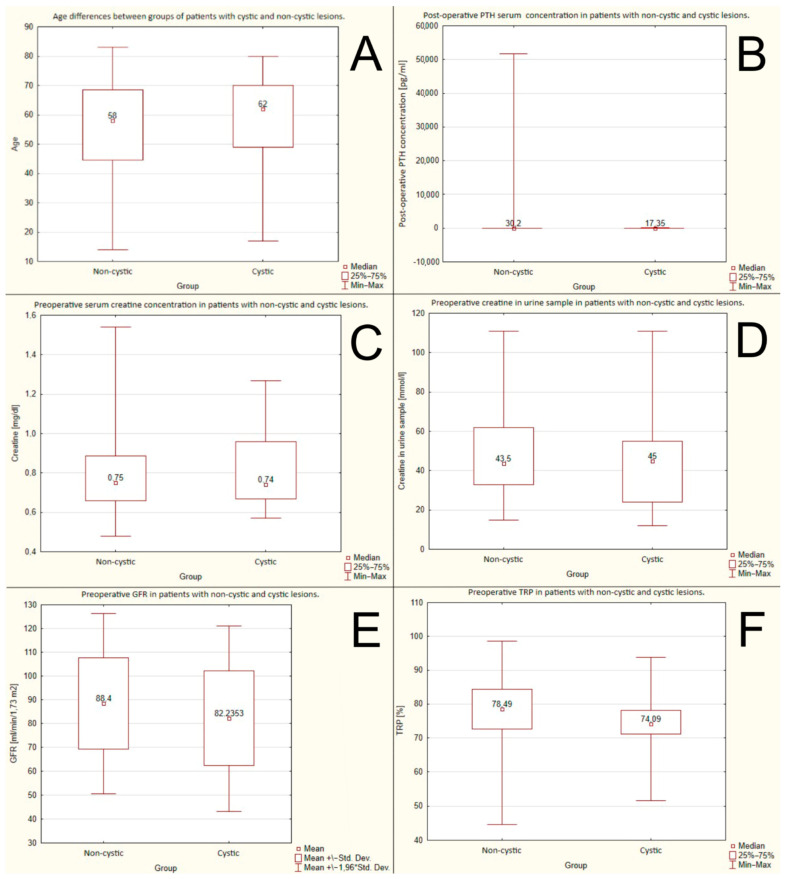
No significant differences between groups of cystic and non-cystic lesions due to: (**A**) age; (**B**) post-operative PTH serum concentration; (**C**) preoperative creatine serum concentration; (**D**) preoperative creatine in urine sample; (**E**) preoperative GFR (glomerular filtration rate); (**F**) preoperative TRP (tubular reabsorption of phosphorus).

There were no statistically significant differences between the groups with non-cystic and cystic lesions. The mean ages were: 58 years vs. 62 years, respectively, *p* = 0,48. The data are shown in Figure 1A.

#### 3.1.2. Postoperative PTH Serum Concentration

There were no statistically significant differences between the groups with non-cystic and cystic lesions. The mean postoperative PTH serum concentrations were: 30.2 pg/mL or 3.2 pmol/L vs. 17.35 pg/mL or 1.83 pmol/L, respectively, *p* = 0.108. The data are shown in Figure 1B.

#### 3.1.3. Preoperative Serum Creatine Concentration

There were no statistically significant differences between the groups with non-cystic and cystic lesions. The mean preoperative serum creatine concentrations were: 0.75 mg/dL or 66.3 μmol/L vs. 0.74 mg/dL or 65.4 μmol/L, respectively, *p* = 0.66. The data are shown in Figure 1C.

#### 3.1.4. Preoperative Creatinine in the Urine Sample

There were no statistically significant differences between the groups with non-cystic and cystic lesions. The mean creatinine levels in the urine samples were: 43.5 mmol/L vs. 45 mmol/L, respectively, *p* = 0.37. The data are shown in Figure 1D.

#### 3.1.5. Preoperative GFR (Glomerular Filtration Rate)

There were no statistically significant differences between the groups with non-cystic and cystic lesions. The mean preoperative GFRs were 88.4 mL/min/1.73 m^2^ vs. 82.24 mL/min/1.73 m^2^, respectively, *p* = 0.227. The data are shown in Figure 1E.

#### 3.1.6. Preoperative TRP (Tubular Reabsorption of Phosphorus)

There were no statistically significant differences between the groups with non-cystic and cystic lesions. The mean preoperative TRP rates were: 78.48% vs. 74.09%, respectively, *p* = 0.15. The data are shown in Figure 1F.

### 3.2. Statistical Differences between the Groups with Non-Cystic and Cystic Lesions

#### 3.2.1. Volume of the Parathyroid Gland

**Figure 2:**
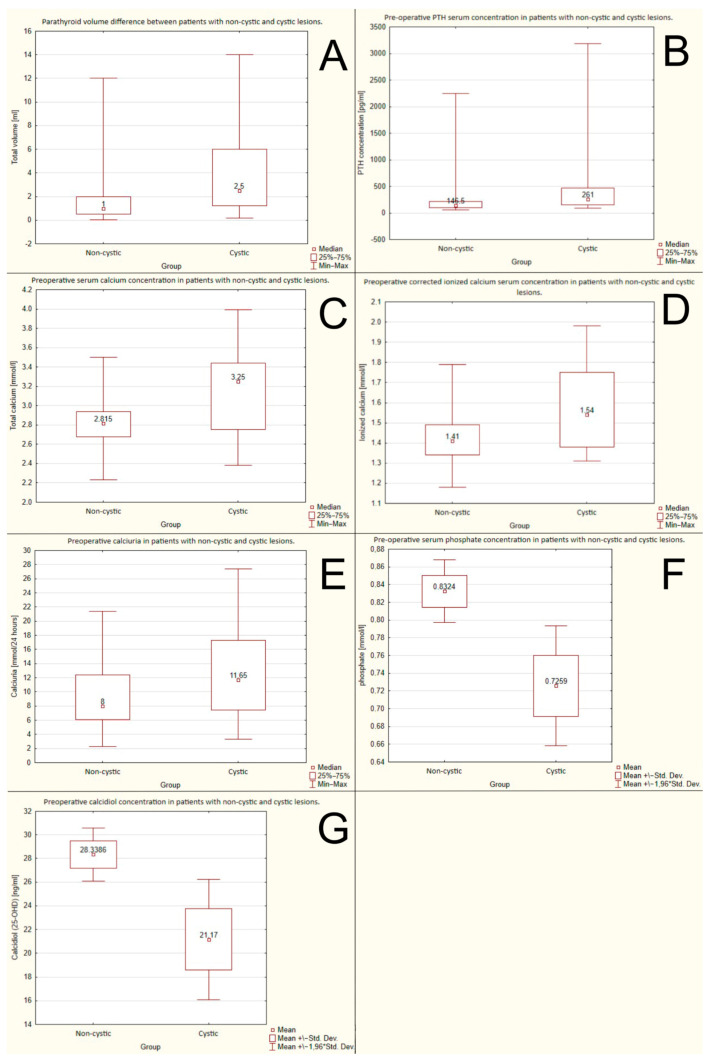
Differences between groups of cystic and non-cystic lesions due to: (**A**) volume; (**B**) preoperative PTH (parathormone) serum concentration; (**C**) preoperative serum calcium concentration; (**D**) preoperative ionized calcium serum concentration; (**E**) preoperative calcinuria; (**F**) preoperative serum phosphate concentration; (**G**) preoperative calcidiol.

The cystic lesions were significantly larger than non-cystic ones. The mean volumes were: 2.5 mL vs. 1 mL, respectively, *p* = 0.0055. The data are shown in Figure 2A.

#### 3.2.2. Preoperative PTH Serum Concentration

Preoperative PTH serum concentration was significantly higher in the group with cystic lesions. The mean PTH serum concentrations were 261 pg/mL (27.67 pmol/L) vs. 146.5 pg/mL (15.53 pmol/L), respectively, *p* = 0.0011. The data are shown in Figure 2B.

#### 3.2.3. Volume of the Parathyroid Gland in Relation to PTH Concentration

**Figure 3:**
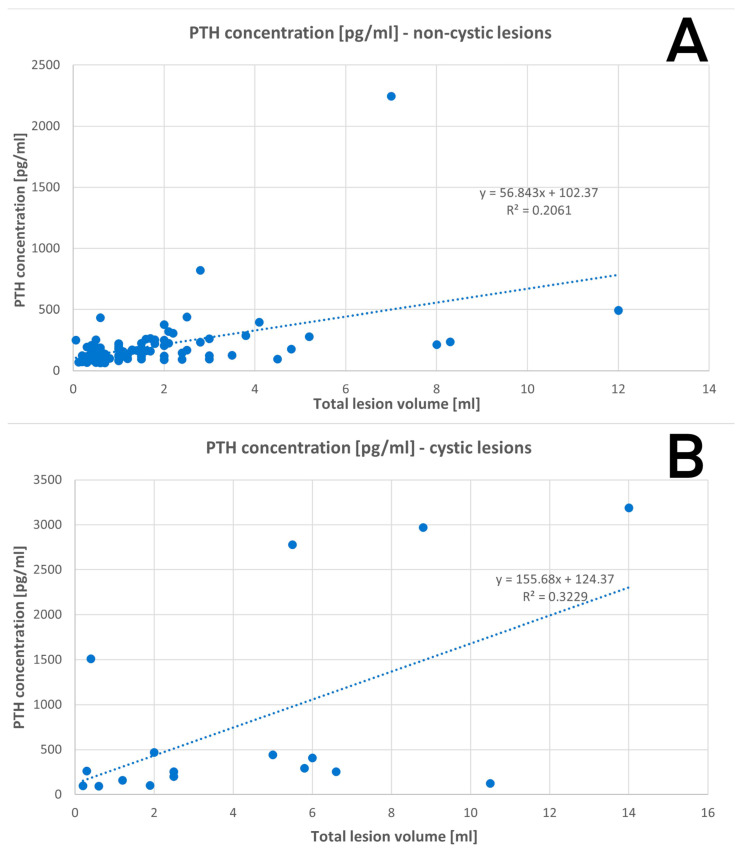
Regression: (**A**) PTH concentration in non-cystic lesions; (**B**) PTH concentration in cystic lesions.

Cystic parathyroid lesions were related to a higher PTH serum concentration in relation to non-cystic lesions of comparable volume. The data are shown in Figure 3A,B.

#### 3.2.4. Total Preoperative Serum Corrected Calcium Concentration (Adjusted to the Albumin Serum Level)

The preoperative serum calcium concentration was significantly higher in the group with cystic lesions than in the non-cystic ones: 3.25 mmol/L vs. 2815 mmol/L, respectively, *p* = 0.0077. The data are shown in Figure 2C.

#### 3.2.5. The Preoperative Corrected Ionized Calcium Serum Concentration

The preoperative ionized calcium serum concentration was higher in the group with cystic lesions than in the non-cystic ones: 1.54 mmol/L vs. 1.41 mmol/L, respectively, *p* = 0.019. The data are shown in Figure 2D.

#### 3.2.6. The Preoperative Calcinuria

Higher values of calcinuria were observed in the group with the cystic lesions than in the non-cystic one: 11.65 mmol/24 h vs. 8 mmol/24 h, respectively, *p* = 0.042. The data are shown in Figure 2E.

#### 3.2.7. Preoperative Serum Phosphate Concentration

Preoperative phosphate serum concentration was higher in patients with non-cystic lesions than in those with cystic ones: 0.832 mmol/L vs. 0.725 mmol/L, *p* = 0.022. The data are shown in Figure 2F.

#### 3.2.8. Preoperative Calcidiol Concentration

Higher calcidiol serum levels were observed in patients with non-cystic lesions than in those with cystic ones: 28.34 ng/mL (70.7 nmol/L) vs. 21.17 ng/mL (52.83 nmol/L), respectively, *p* = 0.0199. The data are shown in Figure 2G.

**Figure 4:**
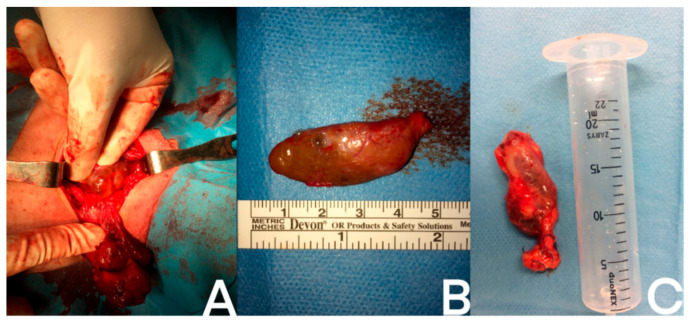
Examples of different cystic parathyroid lesions: (**A**) intraoperative photo of the cystic parathyroid gland; (**B**) removed cystic parathyroid gland; (**C**) removed cystic parathyroid after measuring its volume. A ruler and a syringe serve as reference to show the size of the glands.

A parathyroid cystic lesion visualized during surgery is presented on the Figure 4.

**Figure 5:**
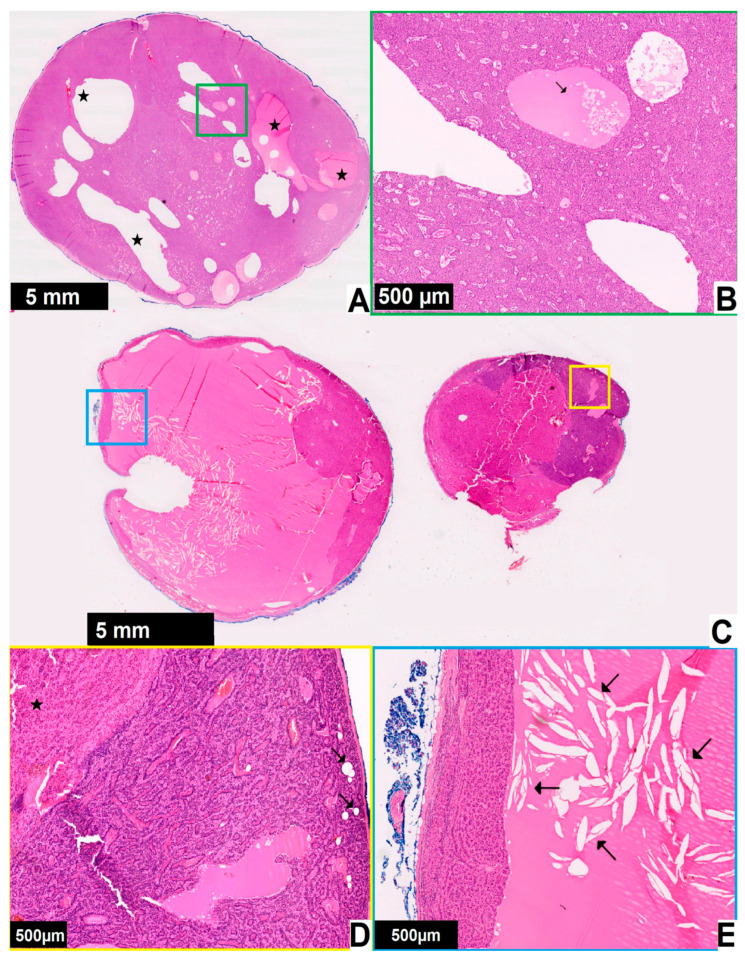
Pathomorphological images in two patients with cystic parathyroid glands ((**A**,**B**)—patient 1, (**C**–**E**)—patient 2). Black magnification bar in the bottom left corner represents the corresponding 5 mm or 500 μm distance on each image. The enlarged areas are marked with the frames of corresponding colors: (**A**) parathyroid gland from a patient with primary hyperparathyroidism and enlargement of a single gland, no fat tissue is present and cyst formation (star) can be seen; (**B**) at higher magnification some cystic spaces are filed with eosinophilic material with a few chief cells within it (arrow), the case was diagnosed as parathyroid adenoma with cyst formation; (**C**) one of the parathyroid glands from a patient with primary hyperparathyroidism, the growth pattern is nodular; (**D**) higher magnification shows the lesion (star) and parathyroid parenchyma with remaining fat cells (arrows); (**E**) there is a large cystic space with eosinophilic material and cholesterol clefts (arrows), the case was diagnosed as parathyroid nodular hyperplasia with cystic degeneration.

Pathomorphological images in patients with cystic parathyroid glands can be seen in Figure 5.

## 4. Discussion

One of the most common endocrinological pathologies is primary hyperparathyroidism (PHPT), caused by excessive secretion of PTH from one or more parathyroid glands. It is characterized by hypercalcemia—chronic increase in calcium serum concentration that is two standard deviations above the mean values (greater than 2.6 mmol/L, 10.4 mg).

Up to 90% of PHPT is caused by sporadic parathyroid adenoma (respectively, solitary—80%, multiple adenomas—5%). Multiple gland hyperplasia accounts for 10–15% of cases and parathyroid cancer—less than 1% [15,16,17,18].

Prevalence of PHPT varies between 0.4 to 82 cases per 100,000 [18]. PHPT frequency increases with age, and this condition is most common in women (with a gender ratio of 3:1) and African Americans. It is mainly diagnosed in the fifth and sixth decade of life. In the younger population, other causes (mostly genetic) are involved, such as multiple endocrine neoplasms type 1, 2 and 4 (MEN1, MEN2, MEN4), hyperparathyroidism-jaw tumor syndrome (HPT-JT). Other reasons for PHPT included familial isolated hyperparathyroidism, familial hypocalciuric hypercalcemia (FHH) and severe neonatal hyperparathyroidism [17,18].

Cystic parathyroid adenomas are uncommon causes of PHPT (less than 1–2%). In the literature, from 19th century, only less than 400 cases of parathyroid cysts were reported, with the first one described by Sandstrom in 1880 [9,19,20,21].

Due to the rare occurrence of parathyroid cysts, the data available in the literature were mainly limited to case reports and small case series of non-functional ones, mostly in the radiological journals. Recently a large meta-analysis with a systematic review was published. The authors used the PubMed and Cochrane databases to search for articles that were published between 1995 and 2020. The review gathered up to 39 studies with a total of 160 patients, diagnosed mainly with a single cystic adenoma [6].

Our study analyses a group of as many as 17 patients with hyperparathyroidism and parathyroid cysts. To the authors knowledge this is one of the largest groups of functional parathyroid cysts ever gathered and reported. It may be an additional source of data for this scarce condition.

Similarly to other endocrine diseases, parathyroid cystic adenomas are encountered more frequently in women with a female-to-male ratio of 2:1 [9,19,20,21]. In the literature concerning PHPT, the gender ratio oscillates to about 3:1 [2,4].

In our study group females accounted for 86.7% (15/17), while males accounted for 13.3% (2/17) of patients, with a gender ratio of 7.5:1. In the control group there were 88 women and 12 men, which gives the gender ratio of 7.3:1.

The average age of the patient with parathyroid cystic lesion was 58. Most patients were diagnosed between the fourth and seventh decade of life with one patient in their second decade. This is consistent with the literature data, which informs that the most common age for making the diagnosis of PHPT is in the fifth and sixth decade of life [2,4].

In the literature, various parathyroid cysts’ etiologies can be found. One of them implies cysts have an embryologic origin from the remnants of the 3rd and the 4th brachial pouches. Moreover, another theory suggests that parathyroid cysts can be formed by enlargement or fusion of several parathyroid microcysts. A recurring thesis assumes that initiation of the creation of the functional parathyroid adenomas could be degeneration, hemorrhage or infarction of a parathyroid gland or adenoma [19,20].

Parathyroid cysts can be divided, based on their ability to secrete hormones, into non-secreting or non-functional (85–90%) and secreting or functional (10–15%). Non-secreting cysts are 2.5 times more frequently encountered in females, while secreting cysts are 1.6 times more common in males. Both types are characterized by highly elevated PTH levels in the cyst fluid, however, increased PTH serum levels were described only in secreting lesions [10,21].

Despite the higher incidence of non-functioning cysts observed in the literature, our team did not encounter such cases. This may be due to the specificity of the endocrine surgery ward—most of the patients admitted here presented features of hyperparathyroidism.

Our study group consisted of 17 patients with functional parathyroid cysts, which were 7.5 times more frequent in females. These discrepancies may be due to the small size of the study group, however, it is still one of the biggest, and to the authors knowledge, the largest cohort of secreting parathyroid cysts ever collected in a single center study. With such a rare condition, collecting a larger group may only be possible by multicenter cooperation.

Most cases, especially those caused by non-functional cysts, are diagnosed incidentally during imaging examination (ultrasound—US, computed tomography—CT, single-photon emission computed tomography-computed tomography—SPECT-CT) [6,10,20,21]. In our patients the US and SPECT-CT were performed.

Some of the symptoms of cystic parathyroid lesions may result from the effect of mass, which may be caused by giant cysts. Then, hoarseness (due to vocal cord paresis), dysphagia, pain, neck mass or dyspnea (following tracheal deviation), thrombosis of innominate veins or recurrent laryngeal nerve paralysis have been reported [10,21]. In our patients there were no symptoms of the effect of mass or compression.

In patients with secreting cysts, symptoms related to hypercalcemia may be observed. They depend on the serum calcium concentration and on the pace of its increase. Due to the development of diagnostic methods and their increasing availability, currently up to 80% of cases of hypercalcemia are asymptomatic or oligosymptomatic, especially in mild hypercalcemia (with serum total calcium up to 3.0 mmol/L or 12 mg/dL). The classic symptoms of hypercalcemia, dominating in the past were nephrolithiasis, osteoporosis, gastrointestinal disorders, osteoarticular pain and neuropsychiatric symptoms.

Hypercalcemic syndrome may develop in moderate to severe hypercalcemia or rapidly progressing hypercalcemia. In the course of this condition the following symptoms resulting from the disfunction of different internal organs may be observed:

- kidneys (polyuria, hypercalciuria, nephrolithiasis);
- gastrointestinal tract (loss of appetite, dyspeptic symptoms, vomiting, constipation, peptic ulcer disease, pancreatitis, cholelithiasis);
- cardiovascular system (hypertension, arrhythmias, tachycardia, shortening of the QT interval);
- neuromuscular symptoms (decreased muscle strength and tendon reflexes);
- cerebral symptoms (fatigue, drowsiness, headache, depression, coma).

If the blood calcium level exceeds 3.75 mmol/L (15.0 mg/dL), symptoms of hypercalcemic crisis may develop. They include: altered consciousness, weakness, nausea and vomiting, abdominal pain, inflammation of the pancreas, cardiac arrhythmias abdominal pain, polyuria and dehydration [2,21,22,23,24,25,26].

All our patients (from both the study and the control group) presented biochemical features of hypercalcemia. The symptoms resulted from calcemic levels and the duration of the disease—some patients suffered from osteoporosis or nephrolithiasis.

The differential diagnosis of parathyroid cysts should include thyroid gland cyst, branchial cleft cyst, thyroglossal duct cyst, thyroid adenoma and parathyroid carcinoma.

Initial diagnosis is important for the therapeutic process and could help to avoid intraoperative cyst rupture as well as parathyromatosis, which might be its consequence. Often the final diagnosis is made during surgery or postoperative histopathological examination [10,21].

To differentiate cystic from solid lesions, ultrasound is the method of choice, due to its non-invasive character. In order to determine the nature and the origin of the lesion, a fine needle ultrasound-guided biopsy is performed. An elevated PTH concentration proves the parathyroid origin, however, only the blood test differentiates whether the cyst is active or not. In case of solitary neck mass, a biopsy cannot provide a definite diagnosis. Computed tomography (CT) or nuclear magnetic resonance (NMR) should be performed. A notable fact is the occurrence of false negative results when using Tc99-m Sestamibi scintigraphy. Up to 32% of patients lack the uptake of the tracer despite elevated PTH concentration in the cystic fluid. The uptake of the tracer depends on the number of parathyroid cells in the cystic wall.

The examination that confirms the diagnosis is the histopathological evaluation of the removed lesion—pathognomonic feature is the presence of parathyroid tissue in the cyst wall [10,19,20,21].

In this study the US and SPECT examinations were performed. In some cases, false-negative results were obtained.

The treatment depends on the character of the lesion. In small, non-functioning cysts ultrasound-guided aspiration or sclerosing therapy using tetracycline or ethanol can be performed. Possible complications of sclerotherapy, such as neurotoxicity or paralysis of the recurrent laryngeal nerve should be considered. For large or functioning cysts, suspicion of malignancy, recurrence of the cyst, or cyst complicated by dysphagia, dyspnea or paralysis of the recurrent laryngeal, surgery should be the first line of treatment. The first sign of the successfulness and completeness of the surgery is the drop in PTH serum concentration. Due to risk of hypocalcemia, postoperative blood calcium concentration should be monitored [10,19,20,21].

All patients from the study and the control group underwent surgical treatment due to symptoms and complications of hypercalcemia. The successfulness of the surgery was confirmed by a decrease in serum PTH concentration. PTH and calcium levels were monitored in each case in the postoperative period.

Due to the rarity of the issue and the limited number of patients, the study has limitations. The study is a retrospective one. In some patients not all the data were accessible, and only the data which were available in all patients could be analyzed. A prospective study would allow for collecting and analyzing a broader range of clinical information.

The volume of the removed glands was precisely measured using Archimedes’ principle, however, the cyst wall was not preserved intact in all cases, so some of the results may be underestimated to a certain extent. The regression analysis showed that the cystic parathyroid lesion relates to a higher PTH serum level than non-cystic lesions of comparable size.

In both our groups there were patients with primary hyperparathyroidism and a suspicion of adenoma. After histological examination it was revealed that there were 71 cases of adenoma (11 patients in the study group and 60 patients in the control group) and 46 cases of hyperplasia (6 patients in the study group and 40 patients and in the control group). Pathological assessment is difficult due to the lack of clear diagnostic criteria for adenoma and hyperplasia, especially in the absence of clear clinical information. A fragment of normal parathyroid tissue is required for the diagnosis of adenoma [27]. Because in some patients it was not possible to preserve an intact cyst, there is a possibility that some cases were mistakenly assessed as hyperplasia. What favors the diagnosis of adenoma is the fact that normalization of PTH was observed in all patients after removal of the only cystic parathyroid gland. The functioning nature of the cyst was also confirmed by the normalization of PTH after removal of the cystic parathyroid gland.

All patients had blood calcium corrected for albumin level (total and ionized) assessed, and there were no cases of renal failure that may interfere with the assessment of PTH, calcemic or urinary electrolyte excretion.

There were no available data regarding vitamin D supplementation and its duration. The analysis revealed that a greater deficiency was found in the group with cystic parathyroid glands, so it can be hypothesized whether vitamin D deficiency may play a role in the formation of parathyroid cysts.

Our study presents one of the largest single center groups with functional cystic parathyroid lesions. The results suggest that parathyroid cystic lesions are associated with an increased risk of severe hypercalcemia. To explore the issue further, a multicenter cooperation is required.

## 5. Conclusions

Cystic parathyroid lesions are associated with higher levels of serum PTH and calcium than non-cystic lesions of similar volume. Therefore, they can be considered as independent risk factors for severe hypercalcemia.

Patients with parathyroid cysts should receive more frequent and thorough medical care.

Due to the increased risk of severe hypercalcemia in patients with parathyroid cystic lesions, earlier surgical treatment may be considered.

Because of the rarity of the phenomenon, further multicenter studies are required.

## Figures and Tables

**Table 1:** The detailed data concerning study and control groups.

	All	Non-Cystic	Cystic
Number of patients	117	100	17
Male	14	12	2
Female	103	88	15
Average age (y.o., years old)	56 y.o.	55 y.o.	58 y.o.

## Data Availability

Data sharing not applicable.
